# Degree of Conversion and Oxygen-Inhibited Layer Effect of Three Dental Adhesives

**DOI:** 10.3390/dj4040037

**Published:** 2016-10-27

**Authors:** Lindsay Robertson, Melissa Phaneuf, Asmaa Haimeur, Igor Pesun, Rodrigo França

**Affiliations:** 1 Dental Biomaterials Research Lab, College of Dentistry, University of Manitoba, Winnipeg, Manitoba R3E0W2, Canada; robertson_lindsay@hotmail.com (L.R.); melissa.j.phaneuf@live.ca (M.P.); 2 Department of Restorative Dentistry, College of Dentistry, University of Manitoba, Winnipeg, Manitoba R3E0W2, Canada; Asmaa.Haimeur@umanitoba.ca (A.H.); Igor.Pesun@umanitoba.ca (I.P.)

**Keywords:** self-etch adhesives, degree of conversion, oxygen-inhibited layer

## Abstract

This study investigated the effect of the oxygen-inhibited layer on the degree of conversion (DC) of three dental adhesives, comparing two different protocols. Quartz–tungsten–halogen (QTH) light curing and light-emitting diode (LED) were used to cure three adhesives: OptiBond All in One (OAIO), Adper Easy Bond (AEB) and ExciteF (EXF). The DC was calculated utilizing Fourier Transform infrared spectroscopy (FTIR) (n = 12). The two protocols used were as follows: (i) prevent the oxygen-inhibited layer using a Mylar plastic strip pushed onto each bonding adhesive; and (ii) polymerize samples without a plastic strip. The data was analyzed statistically by a three-way ANOVA, and Tukey Test (a = 0.05). The presence of an oxygen-inhibited layer reduced the DC of the adhesives by 64% for EXF, 46% for AEB and 32% for OAIO. This study suggests that there are differences among the oxygen-inhibited layers present for the adhesives tested.

## 1. Introduction

Dental bonding adhesives are important materials in dentistry for bonding both direct and indirect restorative materials to enamel and dentin [1]. Many generations of dentin bonding adhesives exist, and each new generation brings advancements in bonding adhesive technology [1,2]. Dentin bonding adhesives contain resin monomers that need to be converted into polymers; in an ideal situation, all monomers should be transformed into a polymeric chain, but this does not happen. The ratio of the amount of monomers converted in a polymer is called the degree of conversion (DC). Currently, the majority of dentin bonding adhesives require visible light curing to perform this polymerization. In the past, the most common curing device was the quartz–tungsten–halogen (QTH) light, but since the 1990s; light-emitting diodes (LED) have become an alternative that is commonly used as a curing device [3]. It has been shown that some new adhesive initiators have better polymerization when they are QTH- or LED-activated [4].

The DC has been used to assess the effectiveness of polymerization in dentin bonding adhesives for in vitro studies [3,4]. A high DC is desirable, but there are factors that contribute to lowering the DC [4,5,6,7,8,9,10]. After being cured, bonding adhesives contain an unpolymerized superficial layer because of contact between the initiators and oxygen from the atmosphere [11,12]. This gel-like surface, also known as the oxygen-inhibited layer (OIL), has been suggested to inhibit polymerization and contribute to lowering the DC [12]. However, because previous results have been controversial, the OIL requires further evaluation.

Several studies have used the DC of dental adhesives as a parameter for predicting mechanical properties and long-term behavior [4,8,9,10]. The most common protocol for DC calculation consists of comparing an unpolymerized drop of adhesive with a polymerized one using infrared or Raman spectroscopy. During the in vitro preparation of the polymerized samples, a polyester strip is placed on top of the adhesive drop before light curing to prevent oxygen from reacting with the adhesive components and subsequent OIL formation. However, under clinical conditions, it is impossible to avoid OIL formation. In the clinical protocol (in vivo) after the adhesive coat is applied onto the enamel and/or dentin, the dentist light-cures it directly and there is no barrier to prevent contact between the adhesive and oxygen.

This study analyzes the DC of two self-etch adhesives comparing them with one total-etch adhesive using Fourier Transform infrared spectroscopy (FTIR). Each adhesive was cured using one of two different light curing devices: a QTH curing light or a LED curing light. Also, this study investigated the effect of the oxygen-inhibited layer on the DC of these adhesives, comparing two different protocols. The null hypotheses tested were: (1) there is no difference between the DC among these three adhesives; (2) the DC of the adhesives does not differ according to light curing activation device; and (3) the DC is not affected by the in vitro protocol compared with the in vivo protocol (no OIL displacement).

## 2. Results

**Figure 1:**
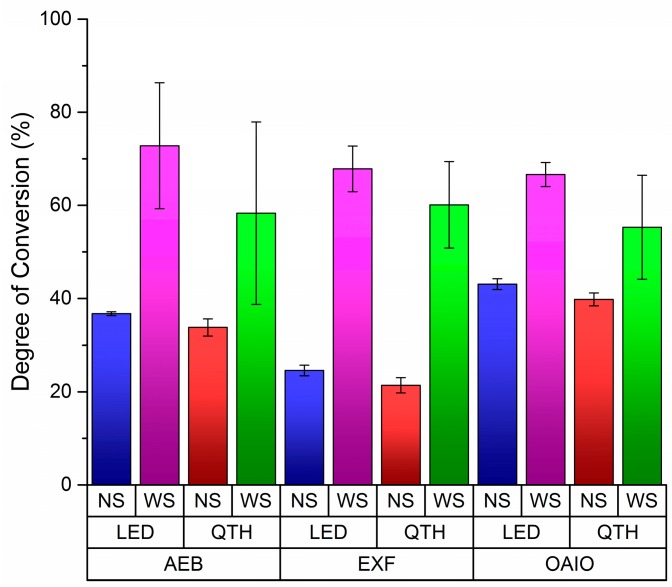
Degree of conversion for each brand of dental adhesive using different curing techniques. Three adhesives were used: Adper Easy Bond Self-Etch Adhesive (AEB), ExciteF Vivapen (EXF), OptiBond All-In-One (OIAO). LED and QTH are the two light curing techniques used. No strip (NS) and with strip (WS) were the two protocols to measure the effect of oxygen- inhibited layer.

Table 1 shows the average percent DC and the standard deviations (SD). The adhesive brand factor was not statistically significant (*p* = 0.06), but the light curing device was significant (*p* = 0.015). The DC values from the interaction between the bonding agent and light curing was not significant. The protocol, which indicates the effect of OIL was strongly significant (*p* = 7.56 × 10^−7^). The interaction among the three factors (adhesive brand, bonding agent, and light curing) was not significant (*p* = 0.87); however, as shown in Figure 1, Tukey’s post-hoc test has indicated that some means are significant at alfa level (0.05%).

**Figure 2:**
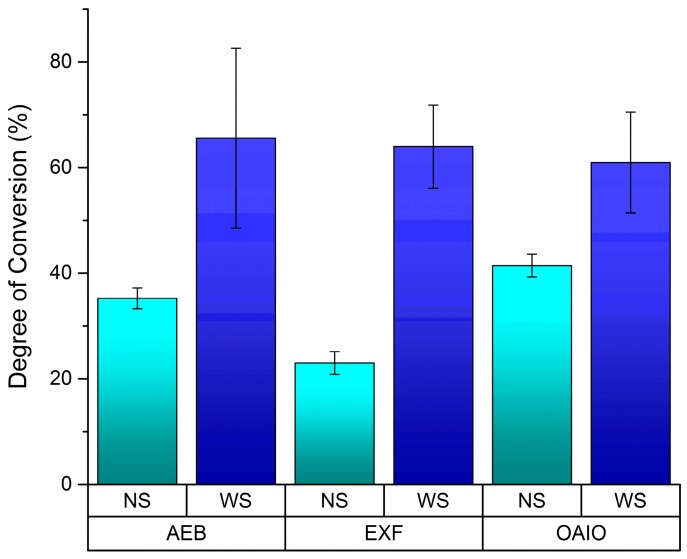
Degree of conversion for the interaction between bonding agents and the technique for the oxygen-inhibited layer (OIL) displacement.

**Figure 3:**
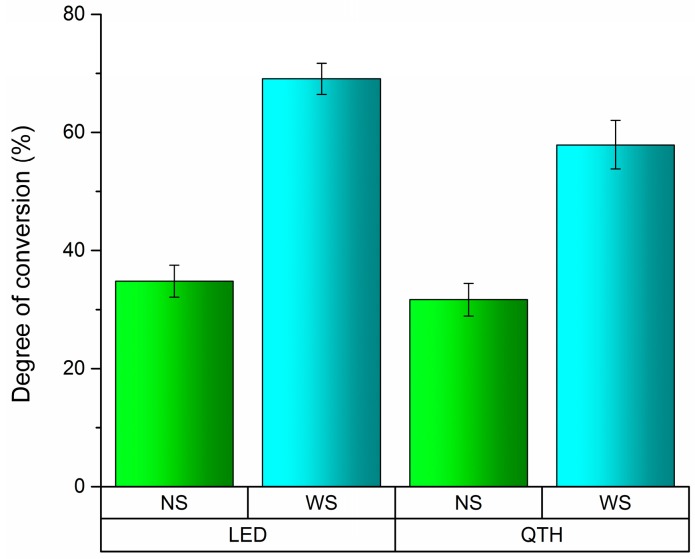
Means and standard deviation (SD) of the degree of conversion for the interaction between light curing and protocol of OIL displacement.

Without OIL displacement (no strip (NS)), OptiBond All in One (OAIO) had the highest DC values for every kind of light curing system. In the same condition, ExciteF (EXF) had the lowest DC values for every category in the light curing system, and the values for Adper Easy Bond (AEB) were in between those for OAIO and EXF. In Figure 2, EXF showed the biggest difference between the NS and with strip (WS) protocols, which may indicate that oxygen has the greatest effect on EXF’s polymerization system. Also, Tukey’s post-hoc test indicated that there was a difference between LED and QHT when OIL was displaced (*p* = 0.008), as shown in Figure 3.

## 3. Discussion

The results of this study showed that there was no statistical difference among the DC of the tested bonding agents, accepting the first null hypothesis. The effect of light curing and protocol on the DC were statistically significant, and thus the second and third hypotheses were rejected.

Ideally, light curing of resin-based materials would result in a completely polymerized sample with no unpolymerized material remaining [13,14,15,16,17,18,19]. However, even when using experimental adhesives, the highest DC values reached we Please explain each mark re around 80% [20,21]. A low DC can lead to low mechanical properties and more permeability [17,22]. Also, a large amount of unpolymerized acid monomers in self-etch adhesives could result in a continuous etching process of dentin [21]. It is important to consider that composite resin-based filling materials undergo polymerization shrinkage. These contractile forces will be the most concentrated at the interface between the composite resin and the dentin adhesive. If the underlying dentin adhesive is poorly cured, it will not be strong enough to withstand these contractile forces, and cohesive lines of fracture may occur in the adhesive creating a gap between the dentin adhesive and composite resin, which could eventually cause clinical failure of the composite resin restoration. A low DC could facilitate the propagation of these cohesive lines [17].

In this study, there was no difference between the DC among the three adhesive tested. However, other studies have shown different results [3,4,8,10]. These differences can be explained by the technique used in each study. For example, in this study the manufacturer’s recommendations related to solvent evaporation time and light curing time (only 10 s) were followed. A different result would be obtained if double the light curing time was used [4]. Also, a longer distance (1 cm) from the tip of the light curing unit to the adhesive was chosen in this study to better represent the clinical reality. This decision may influence the results of the interaction between the bonding agent and light curing.

It is well established that OIL interferers in the polymerization reaction, lowering DC values [11]. Thus, the DC standard test protocol recommends to use a plastic Mylar strip placed on the samples during light curing to displace the oxygen present on the surface [4,8,10,22]. It is accepted and suggested that a small amount of monomers remain uncured to allow adhesion to the filling material placed on top. According to current clinical understanding, if the surface layer of the dentin adhesive is initially unpolymerized, it can potentially allow the composite resin placed on top to flow into the dentin adhesive, creating an adhesive/resin zone that will then become polymerized together when the composite resin is light cured [11,12]. However, as demonstrated in this study, the protocol of using a Mylar strip inflates the DC value of the tested adhesives. Table 1 shows that the overall DC averages of the adhesives can be lowered by approximately 50% when no strip is used. This indicates the importance of this finding because, in the clinical situation, no strip is used on top of the adhesive before polymerization. As shown in Figure 3, less than 40% of the adhesive monomers became polymers when no strip was used. Further investigations should be done to verify if a large amount of monomer can become completely polymerized during the light curing process of the composite resin restoration. In this direction, it would be more realistic to consider the results from the NS group than the results from the WS group for each adhesive brand.

The effect of oxygen on the equilibrium of dental bonding agent initiators has already been well explained [12,23]. An important piece of information found in this study is the possibility to measure the effect of the OIL on different bonding agents. Figure 2 illustrates this impact of the OIL on DC values. EXF seems to be the most affected by oxygen during its polymerization. The DC value when EXF is exposed to oxygen (NS) is 64% lower than when a strip was used. This reduction was smaller for AEB (−46%) and OAIO (−32%). Further considerations of the reason for this effect are difficult because the manufacturer of EXF does not disclose what kind of photo-initiator has been used in this adhesive, which is different from AEB and OAIO, which indicate that camphorquinone is part of their composition.

## 4. Materials and Methods

The adhesives and curing devises used in this experiment are shown in Table A1 and sample preparation is presented in Table A2 (n = 12).

This study compares the effects of QTH light curing versus LED curing of dentin bonding adhesives. It also looks at the effect of displacing the oxygen-inhibited layer on the surface of the dentin bonding adhesives while curing (in vitro protocol), versus curing the dentin bonding adhesives while the surface is exposed to air, which is the case in current dental clinical practice (in vivo protocol).

Each sample was prepared according to the manufacturer’s instructions (Table A1) for use of the dentin bonding adhesives in a direct restoration. For half of the samples, an 8-mm plastic Mylar strip (Hawe-Neos Dental, Bioggio, Switzerland) was pushed onto each bonding adhesive sample, displacing the oxygen present on the surface. The plastic strip remained on top of the bonding adhesive until light curing was complete.

The intensity of the QTH and LED curing lights was measured using a radiometer (Model LED, Dementron Research Corporation, NY, USA). The intensity of the curing lights at a distance of 1 cm was recorded.

The prepared samples underwent one of the following procedures:

Cured with the QTH light with no strip (NS)

Cured with the LED light with no strip (NS)

Cured with the QTH light with a plastic strip (WS)

Cured with the LED light with a plastic strip (WS)

### Fourier Transform Infrared (FTIR) Analysis

FTIR (Nicolet 6700, ThermoFisher, Waltham, MA, USA) was performed on each adhesive (n = 12). Samples were underwent 32 scans between the 4000 and 400 cm^−1^ frequency range, with 2 cm^−1^ resolution. Spectra were obtained in the absorbance mode. OMNIC^TM^ Spectra software was then used to add a baseline correction for each spectrum. The degree of conversion of each sample was calculated utilizing the FTIR C = C (1640 cm^−1^) and C = O (1720 cm^−1^) ratio between the polymerized and non-polymerized samples, using the equation below:

(1) $$DC\;=\;1-\;\frac{\mathrm{Area}\;\mathrm{of}\;\mathrm{band}\;C\;=\;C\;\left(\mathrm{polymer}\right)\;/\;\mathrm{area}\;\mathrm{of}\;\mathrm{band}\;C\;=\;O\;\left(\mathrm{polymer}\right)\;}{\mathrm{Area}\;\mathrm{of}\;\mathrm{band}\;C\;=\;C\;\left(\mathrm{monomer}\right)\;/\mathrm{area}\;\mathrm{of}\;\mathrm{band}\;C\;=\;O\;\left(\mathrm{monomer}\right)}\;\times \;100$$

The data were analyzed statistically using a three-way ANOVA followed by Tukey’s post-hoc test (α = 0.05), and the three tested factors were: adhesive brand, light-curing device, and protocol (with strip and no strip).

## 5. Conclusions

This study suggests that there are differences among the oxygen-inhibited layers present for the three dental adhesives tested. It was also clear that the in vitro protocol results (displacing the oxygen present on the surface using a Mylar strip) can overestimate DC of all adhesives when compared to clinical protocol results (curing the samples without a Mylar strip). Consequently, the DC results using a strip do not reflect the clinical effect of OIL in dental adhesives. The source of light curing was significantly different when all other experimental factors were excluded. Further investigations will be necessary to determine the ideal size of an oxygen-inhibited layer.

## Figures and Tables

**Table 1:** Means and SD of the degree of conversion according to the light sources and curing protocols.

Degree of Conversion (%)				
Adhesives	QTH		LED	
	No Strip	with Strip	No Strip	with Strip
OAIO	39.80 (±1.4) ^a^	55.29 (±11.2) ^a^	43.08 (±1.2) ^a^	66.62 (±2.6) ^a^
AEB	33.79 (±1.8) ^b^	58.31 (±19.6) ^a^	36.73 (±0.4) ^b^	72.8 (±13.5) ^a^
EXF	21.41 (±1.65) ^c^	60.1 (±9.2) ^a^	24.59 (±1.1) ^c^	67.8 (±4.8) ^a^

^a,b,c^ Identical letters in same column did not differ by Tukey’s test (*p* > 0.05).

## Appendix A

**Table A1:** Bonding adhesives and curing light units used.

Brand (Manufacturer)	Abbreviation	Composition	Curing Time (Seconds)
3M ESPE Adper Easy Bond Self-Etch Adhesive (3M ESPE, St. Paul, MN, USA)	AEB	2-hydroxyethyl methacrylate (HEMA), Bis-GMA, Methacrylated phosphoric esters, 1,6 hexanediol dimethacrylate, Methacrylate functionalized Polyalkenoic acid (Vitrebond™ Copolymer), silica filler with 7 nm primary particle size, Ethanol, Water, Initiators based on camphorquinone, Stabilizers	10
OptiBond All-In-One (Kerr Corporation, Orange, CA, USA)	OAIO	Glycerol phosphate dimethacrylate and di-functional methacrylate monomers, water, acetone and ethanol, Photo-initiator—camphorquinone based 3 nm-sized fillers, Fluoride-releasing fillers—sodium hexafluorosilicate and ylterbium fluoride	10
ExciteF Vivapen (Ivoclar Vivadent, Amherst, NY, USA)	EXF	phosphonic acid acrylate, Bis-GMA, ethanol 2-hydroxyethyl methacrylate, urethane dimethacrylate, SiO^2^, potassium fluoride, initiators and stabilizers	10
Light Source	Supplier	Type	Intensity
Quartz-Tungsten-Halogen (QTH)	Dentsply	The Max_Caulk	450 mw/cm^2^
Light-Emitting Diode (LED)	Ultradent Products Inc.	Valo Cordless	900 mw/cm^2^

**Table A2:** Sample preparation according to manufacturer’s recommendations.

Bonding Adhesive	Sample Preparation
**AEB**	- Applied a thin layer of the adhesive to an IR slide using a microbrush (unidose packaging). - Allowed 20 s for the adhesive to remain exposed to the air to allow the solvent to evaporate. - Used an air syringe for 5 s, providing a gentle stream of air. - Light cured for 10 s.
**OAIO**	- Shook bottle of adhesive (unidose packaging). - Applied a thin layer of adhesive to aluminum using a microbrush. - Allowed 20 s for the adhesive to remain exposed to the air to allow the solvent to evaporate. - Used an air syringe for 5 s, providing a gentle stream of air. - Light cured for 10 s.
**EXF**	- Removed pen cap and inserted the VivaPen brush tip - Pressed the click button to saturate the VivaPen brush tip with Excite F bonding agent (when the tip became yellow). - Applied a thin layer of bonding agent to an infrared slide using the brush tip. - Allowed 10 s for the bonding agent to remain exposed to air to allow solvent to evaporate. - Used an air syringe for 5 s, providing a gentle stream of air. - Light cured for 10 s.
